# Multiple Epidermal Inclusion Cysts of the Female Breast Coexisting With Fibroadenoma: A Case Report of an Incidental Finding

**DOI:** 10.7759/cureus.52832

**Published:** 2024-01-23

**Authors:** Babatope L Awosusi

**Affiliations:** 1 Pathology and Laboratory Medicine, King Khalid Hospital, Al Majmaah, SAU

**Keywords:** histopathologic evaluation, keratin, breast lump, fibroadenoma, epidermal inclusion cyst

## Abstract

This report discusses a case of a 27-year-old female with histopathological examination results of multiple epidermal inclusion cysts (EICs) coexisting with fibroadenoma without any involvement of breast skin. EIC should be considered as one of the differential diagnoses for benign breast lesions. Radiological evaluation, with surgical excision and histopathologic examination, remains the gold standard for the management of EIC. This report aims to increase the level of awareness on the existence of EIC in the breast parenchyma and the possibility of it coexisting with or arising from other benign breast lesions like fibroadenoma.

## Introduction

An epidermal inclusion cyst (EIC) is a common benign skin lesion comprising a cyst cavity lined by keratinizing stratified squamous epithelium with the lumen containing keratin and lipid-rich debris [1]. It is usually located in the dermis or subcutaneous tissue. [1]. EICs are one of the three types of epidermal cysts, and the other two include trichilemmal cysts and sebaceous cysts; they can be differentiated by their lining epithelia and associated adnexa [2]. The sebaceous cyst has the same epithelial lining as the EIC but with associated skin adnexal structures (eccrine, pilosebaceous, and apocrine glands) while the trichilemmal cyst is lined by pseudostratified squamous epithelium showing abrupt keratinization without a layer of granular cells [2].

Though EICs can occur anywhere in the body, they are commonly located in the head and neck region, trunk, and extremities [2]. The presence of EIC in the breast is not common and when it does occur, it is usually located in the peri-areolar region [3].

This report discusses a case of a 27-year-old female with histopathological examination results of multiple EICs coexisting with fibroadenoma of the breast without any involvement of breast skin.

## Case presentation

A 27-year-old female presented with a painless and progressively enlarging right breast lump. There was no previous history of breast lumps or breast pathology. There was also no history of trauma or past breast surgeries.

On examination at her presentation, she was conscious and alert. On breast examination, a firm mobile swelling was noted in the upper outer quadrant of the right breast deep in the posterior half of the breast parenchyma, measuring 4 x 3 cm. It was non-tender with no skin changes. The swelling was not attached to the skin or underlying tissues. Systemic examination was unremarkable.

Ultrasound examination of the breast mass showed features suggestive of fibroadenoma, this was also corroborated by a fine needle aspiration cytology (FNAC). Other laboratory investigations done were unremarkable. The patient had surgical excision of the breast lump and the specimen was sent for routine histopathological evaluation.

**Figure 1:**
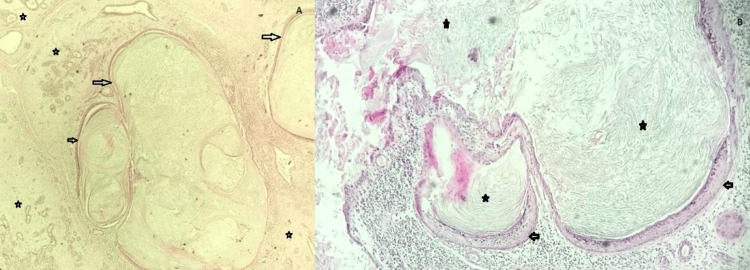
Photomicrograph showing breast tissue with EICs and background fibroadenoma. A: EICs (black arrows) with background fibroadenoma (black stars). Hematoxylin and eosin stain (x100 magnification). B: Higher power view of the EIC showing the stratified squamous epithelial lining and keratin whorls in the lumen (black stars). Hematoxylin and eosin stain (x200 magnification). EIC: epidermal inclusion cyst.

The sample received at histopathologic grossing was a firm grey-white lobulated tissue measuring 4 x 4 x 3 cm in dimension, and cut sections showed homogenous grey-white surfaces with multiple small cyst cavities. Microscopic examination of the breast tissue showed features consistent with fibroadenoma and multiple EICs of variable sizes containing whorls of keratin debris. These EICs were seen embedded within the fibroadenoma mass (Figure 1).

The postoperative follow-up of the patient has been uneventful.

## Discussion

EICs involving the breast parenchyma are uncommon [1-3]. The first histologically diagnosed EIC of the breast was reported at Johns Hopkins Hospital, Baltimore, USA in December 1900 [2], and to date, only 100 cases of EICs have been described in the English literature from different case reports and literature review [1].

Some theories have been proposed to explain the pathogenesis of EICs, including epidermal implantation into breast tissue following trauma, congenital origin secondary to obstruction of hair follicles, and columnar-to-squamous metaplasia in the dilated ducts of fibrocystic disease, fibroadenoma, and phyllodes tumor [4].

Although there was no history of trauma or past surgical procedures done on the breast in this index case, the patient had FNAC as part of the diagnostic work-up, so this may have caused iatrogenic implantation of some epidermal fragments deep into the breast parenchyma. In addition, since the EIC in this index case was seen occurring with fibroadenoma of the breast, it is also plausible that their development may be due to squamous metaplasia in some of the dilated ducts.

On ultrasound examination of the breast, EIC can resemble fibroadenoma, phyllodes tumor, and even mucinous carcinoma [2]. Mammograms and magnetic resonance imaging can also be used as part of diagnostic work-up to exclude malignancy [1]. EIC appears as a well-circumscribed, homogenous density on mammography [5]. Magnetic resonance imaging shows a “fluid‑like signal with variable low-signal components on T2-weighted images, and a peripheral rim enhancement on gadolinium-enhanced images” [3,6]. FNAC and tissue biopsy are also used to evaluate EICs but they are not mandatory if ultrasound imaging is conclusive [1]. In our case, the preoperative diagnosis was fibroadenoma; there was no suspicion of the coexisting epidermal cysts. The incidental diagnosis was made during a routine histopathologic examination of the excised breast lump.

Although EICs are benign lesions, they can rarely transform into squamous cell carcinoma [4]. There is no consensus on the risk of malignant transformation in EIC, while an old study reported that the rate of malignant transformation is up to 19% [7], another more recent study showed that the malignant transformation in the cyst wall epithelium is actually a very rare condition (0.011%) [8]. The other complications that may develop due to EIC include spontaneous rupture with a risk of infection and abscess formation [4].

## Conclusions

EIC of the breast is an uncommon benign lesion with an existing low risk of malignant transformation. It should be considered as one of the differential diagnoses for benign breast lesions. Radiological evaluation, with surgical excision and histologic examination, remains the gold standard for the management of EIC. Hopefully, this case report will increase the level of awareness on the existence of EIC in the breast parenchyma and the possibility of it coexisting with or arising from other benign breast lesions like fibroadenoma.
